# Periodic limb movements in patients with obstructive sleep apnea syndrome

**DOI:** 10.1038/s41598-021-95018-2

**Published:** 2021-07-28

**Authors:** Xiaobo Zhou, Bo Zhou, Zhe Li, Qiao Lu, Shaoping Li, Zhongyin Pu, Fang Luo

**Affiliations:** 1 Department of Psychosomatics, Sichuan Provincial People’s Hospital, University of Electronic Science and Technology of China, Chengdu, China; 2 Chinese Academy of Sciences Sichuan Translational Medicine Research Hospital, Chengdu, 610072 China; 3 Department of Psychiatry, Panzhihua Shengtai Rehabilitation Hospital, Panzhihua, 617000 China

**Keywords:** Medical research, Risk factors

## Abstract

The aim of the study was to assess the factors associated with periodic limb movements during sleep (PLMS) among obstructive sleep apnea syndrome (OSAS) patients and identify the role of PLMS in patients with OSAS. 303 adult patients with OSAS were included in the study. All patients completed physical examination, Epworth sleepiness scale (ESS), and polysomnography. Diagnosis of PLMS was made if the periodic leg movements index (PLMI) was ≥ 15. Chi-square test, ANOVA, univariate and multivariate logistic regression analyses were conducted to identify factors associated with PLMS among OSAS patients. Statistical analyses were performed with SPSS 26.0 for mac. Statistically significant difference was considered if *P* value < 0 .05. Among the 303 adult patients with OSAS, 98 patients had significant PLMS and the other 205 had no significant PLMS. Compared with OSAS patients without PLMS, OSAS patient with PLMS were older, had shorter REM duration and greater apnea–hypopnea index (AHI) (*P* < 0.05). The study suggests that PLMS is a matter of concern among patients with OSAS. A better understanding of the role of PLMS among OSAS patients could be useful in better recognition, intervention and treatment of OSAS.

## Introduction

Obstructive sleep apnea syndrome (OSAS), as a clinical common and frequently-occurring sleep-related respiratory disease, is mainly manifested as apnea and reduced ventilation during sleep, accompanied by symptoms, such as snoring, disordered sleep structure, decreased arterial oxygen saturation at night and daytime sleepiness.

Periodic limb movements during sleep (PLMS) are frequently observed in OSAS patients. These spontaneous and repetitive muscle contractions frequently involve flexion of the toe, ankle, knee and hip^1^. PLMS was considered responsible for sleep fragmentation, and a complaint of excessive daytime sleepiness (EDS) was often regarded as a consequence of PLMS. However, the extent to which PLMS contribute to excessive daytime sleepiness and disturbed sleep structure is controversial^2^, and some researchers consider PLMS simply as a polysomnographic observation without functional significance^3^. Therefore, we conducted the study to explore the role of PLMS in patients with OSAS.

## Results

A total of 98 OSAS patients with PLMS and 205 OSAS patients without PLMS were included in the study. The group of patients with PLMS included 77 males and 21 females, and the mean age was 47.2 ± 12.4 years. The control group included 157 males and 48 females, and the mean age was 43.4 ± 12.2 years.

Univariate regression analyses were performed to ascertain possible factors associated with the existence of PLMS (Table 1). Among these variables, older age, shorter duration of rapid eye movement (REM) and slow wave sleep, greater apnea–hypopnea index (AHI) and arousal index, greater Epworth sleepiness scale (ESS) scoring, and greater snoring times were significantly associated with the existence of PLMS in the univariate model. Multivariate logistic regression analyses revealed that the existence of PLMS was significantly associated with older age (47.2 ± 12.4 vs 43.4 ± 12.2 years, OR = 1.029, 95% CI = 1.007, 1.051), shorter REM duration (63.0 ± 27.2 vs 73.6 ± 29.1 min, OR = 0.990, 95% CI = 0.981, 0.999) and greater AHI (41.2 ± 26.9 vs 28.3 ± 22.5, OR = 1.023, 95% CI = 1.012, 1.033) (*P* < 0.05) (Table 2).

**Table 1:** Univariate analysis of differences between OSAS patients with PLMS and without PLMS.

Variables	Non-PLMS (n = 205)	PLMS (n = 98)	Statistics	*P* value
Age	43.4 (12.2)	47.2 (12.4)	F = 6.659	0.010**
**Gender**				
Male	157 (76.5%)	77 (78.5%)	χ^2^ = 0.149	0.771
Female	48 (23.4%)	21 (21.4%)		
ESS score	8 (5.5)	9.7 (6.4)	F = 5.859	0.016**
Height	166.9 (7.6)	167.7 (7.6)	F = 0.812	0.368
Weight	71.2 (12.4)	73.2 (13.0)	F = 1.596	0.207
BMI	25.5 (3.7)	25.8 (3.5)	F = 0.564	0.453
SBP before sleep	123.1(15.5)	125.2(14.3)	F = 1.368	0.243
DBP before sleep	74.0 (11.6)	76.6 (11.7)	F = 3.005	0.084
SBP awake in the morning	121.8 (15.0)	125.5(16.0)	F = 3.751	0.054
DBP awake in the morning	75.8 (11.2)	78.2(12.3)	F = 2.735	0.099
**Sleep latency (min)**				
N1	9.2(11.7)	12.0 (24.4)	F = 1.859	0.174
N2	17.1(21.7)	20.8 (32.5)	F = 1.382	0.241
N3	72.3(88.9)	84.8(89.7)	F = 1.305	0.254
REM	131.4(73.2)	144.4(90.9)	F = 1.778	0.183
**Sleep duration**				
N1 + N2	398.1 (258.8)	388.5 (70.0)	F = 0.132	0.716
SWS	45.6 (30.7)	30.0 (30.5)	F = 17.240	0.000**
REM	73.6 (29.1)	63.0 (27.2)	F = 9.110	0.003**
TIB	86.9 (10.9)	84.6 (13.4)	F = 2.647	0.105
Arousal index	20.5 (14.3)	26.7 (19.2)	F = 9.911	0.002**
AHI	28.3 (22.5)	41.2(26.9)	F = 18.983	0.000**
Average SPO_2_	95.7 (1.8)	95.5 (2.0)	F = 1.392	0.239
Minimal SPO_2_	80.4 (13.2)	77.3 (12.8)	F = 3.599	0.059
**Snoring**				
Times	176.8 (169.4)	248.2 (238.2)	F = 8.966	0.003**
Duration	68.2 (165.3)	69.2 (75.7)	F = 0.003	0.957
Percentage	12.7 (14.3)	14.5 (15.2)	F = 0.902	0.343
Heart rate (BPM)	74.0 (10.0)	74.6 (10.8)	F = 0.200	0.655

*ESS* Epworth sleepiness scale, *BMI* body mass index, *SBP* systolic blood pressure, *DBP* diastolic blood pressure, *PLMS* periodic limb movements during sleep, *REM* rapid eye movement, *SWS* slow wave sleep, *TIB* time in bed, *AHI* apnea–hypopnea index, *BPM* beat per minute.

**P < 0.05 is statistically significant.

**Table 2:** Multivariate regression analysis of factors with PLMS among OSAS patients.

Variable	B	Exp (B)	95% CI	*P*
Age	0.028	1.029	(1.007, 1.051)	0.011**
REM duration	− 0.010	0.990	(0.981, 0.999)	0.029**
AHI	0.022	1.023	(1.012, 1.033)	0.004**
ESS scoring				0.133
SWS duration				0.062
Arousal index				0.724
Snoring times				0.171

**P < 0 .05 is statistically significant.

## Discussion

Our study found that OSAS patients with PLMS had significantly shorter REM sleep time than OSAS patients without PLMS. Literature about the relationship between PLMS and REM duration were mixed. A previous study reported that the duration of sleep stages did not show significant alteration whether they were associated with PLMS or not^4^. Another study found that the presence of increased PLMS was associated with disruption of REM sleep^5^. In addition, PLMS was usually observed in patients with REM-sleep behavior disorder (RBD)^6,7^. The 6th World Congress on Sleep Medicine reported that PLMS found to be an independent predictor of mortality in a patient cohort study with RBD through a median follow-up of 7.1 years (The 6th World Congress on Sleep Medicine, Seoul, Korea) , proposed a hypothesis that PLMS and RBD may partly share a common pathogenesis impairment of central dopaminergic transmission^6^. Further studies were warranted to test the hypothesis.

Our study found that OSAS patients with PLMS were associated with older age, which was consistent with previous studies. Previous studies showed that age was positively associated with PLMS^8,9^, It has been reported that PLMS can be found also in normal subjects mostly over the age of 40 years^10,11^. In addition, Raffaele Ferri and his colleges found that typical interval of PLMS was approximately 24–28 s before the age of 55 years but at approximately 14–16 s after the age of 65 years^12^.

Our study found that OSAS patients with PLMS were associated with greater AHI. Literature about the correlation between PLMS and AHI were mixed. Some authors considered PLMS simply as a polysomnographic observation without functional significance^3^. In a recent study, researchers pointed out that there might be two types of related leg movement: true periodic ones possibly temporally displaced or paced by the respiratory events, and purely respiratory related ones, possibly AHI-independent markers of OSAS severity, signaling the presence of frequent arousals, a higher proportion of apneas, and respiratory events of longer duration^13^. Further studies were warranted to explore the correlation between PLMS and AHI.

Our study found that blood pressure and heart rate did not correlate with PLMS. Literature about the correlation between blood pressure, heart rate and PLMS were mixed. Some studies reported an increased blood pressure and heart rate in association with PLMS^14–16^. A proposed mechanism that might link PLMS and cardiovascular disease includes increased sympathetic cardiovascular modulation^17^. Some researchers believed that the activation of sympathetic cardiovascular modulation may partly be explained by the co-activation of leg motor and sympathetic neural fibers in the spinal cord^18^. Other studies addressing the relationship between PLMS and hypertension found an association in unadjusted models, but this was lost after adjusting for potential confounders such as age and BMI^19^. The discrepant results in these studies might be partly explained by important methodological differences. including study design, type of population included, type of blood pressure evaluation and other confounding factors^20^.

Our study found no significant correlation between sleepiness and PLMS. The belief that PLMS could cause sleepiness was widely prevalent, and it was expressed in the International Classification of Sleep Disorders^21^. Data from clinical samples may show a higher rate of sleepiness in patients with PLMS, as excessive sleepiness is often a symptom which prompts initial clinical evaluation. However, some studies, including our study, did not find such relationship^22^. The discrepancy of the findings may partly be explained by the fact that sleepiness is a subjective symptom and it is difficult in quantifying sleepiness. Sleepiness is probably not a unitary concept and could reflect essentially different states. Therefore, although ESS could certainly offer valuable information of sleepiness, it may not grasp all aspects of sleepiness^23^.

Our study found that gender was not a risk factor of PLMS among patients with OSAS. According to previous studies, findings about gender differences of PLMS were mixed. Some studies reported that females had higher prevalence of PLMS than males^24^, and that females even had a fourfold higher prevalence of PLMS than males in patients with sickle cell disease^25^. However, some population-based and clinical studies found no gender differences or even a higher prevalence of PLMS in males than females^26,27^. The discrepancy of the findings may partly be explained by the sample population studied. There were large variations between sample population in terms of race, number of participants, age, and OSAS severity.

Limitations of the study need to be pointed out. The patients enrolled in the study were from only one sleep disorder center, which might result in potential sampling bias. Larger, multi-center studies are required to explore the associated factors of PLMS among OSAS patients A better understanding of the role of PLMS among OSAS patients will be useful in better recognition, intervention and treatment of OSAS.

The present studies found that compared with OSAS patients without PLMS, OSAS patient with PLMS were older, had shorter REM duration and greater AHI. The study suggests that PLMS is a matter of concern among patients with OSAS. A better understanding of the role of PLMS among OSAS patients could be useful in better recognition, intervention and treatment of OSAS.

## Methods

### Participants

Patients with a chief complaint of snoring were referred to the sleep disorder center of department of psychosomatics, Sichuan Academy of Medical Sciences & Sichuan Provincial People's Hospital from 2017 to 2019. All patients completed physical examination, ESS and PSG. To be eligible for this study, participants had to be over 18 years old and meet the criteria of OSAS. Exclusion criteria included severe comorbid conditions or medication usage that might influence PLMS. Finally, a total of 303 patients with OSAS were enrolled in the study.

### Questionnaire

The most important scale for the assessment of daytime sleepiness is ESS published in 1991 by Murray Johns^28^. According to the procedure used in other studies^29,30^, the participants were asked to complete a validated Chinese version of the ESS questionnaire^31^ on the night of examination with the guidance of an experienced sleep specialist. It consists of a self-administered questionnaire that investigates the extent of daytime sleepiness specifically and in a very simple manner. There are 8 items in ESS and subjects are asked to rate on a 4-point scale (0–3) his/her chances of dozing in each of 8 different situations that are often encountered in daily life. ESS scores > 10 reflected the presence of extensive daytime sleepiness as it is commonly applied in sleep studies in China^32,33^.

### PSG data collection

The gold standard diagnostic method for OSAS is a full-night PSG^34^. The frequency of episodes of apnea and/or hypopnea per hour of sleep, also known as AHI, as well as the lowest observed oxyhemoglobin saturation during sleep is used as the main criteria for severity assessment.

All patients had to have a minimum of 8 h of monitored sleep in the sleep disorder center. PSG monitoring (Philips Alice Version 6, Netherland) was used for data acquisition. During PSG recording, we monitored brain activity using six electroencephalographic (EEG) placements (F3—A2, F4—A1, C3—A2, C4—A1, O1—A2, O2—A1), muscle tone by chin electromyography (EMG), eye movements by electrooculography (EOG), heart rate by electrocardiography (EKG), oxygen saturation by finger pulse oximeter. The way of measuring respiratory effort is with bands with stretch sensors placed around the chest and/or abdomen. Thermal sensor was used for detecting apnea events and a nasal pressure sensor for detecting hypopnea events.

Snoring sensors was used to assess the main patterns of snoring and their characteristics. Position monitoring was used to record body position change.

Sleep stages and associated events were assessed and analyzed according to the international criteria of the American Academy of Sleep Medicine (AASM)^35^. According to the AASM criteria^35^, hypopnea was defined as ≥ 30% decrease in flow from baseline for at least 10 s with an associated oxygen desaturation of ≥ 3% or an associated arousal. An apnea was defined as more than 90% reduction in airflow for at least 10 s. The onset of a leg movement event is defined as the point at which there is an 8 μV increase in EMG voltage above resting EMG; the ending of a leg movement event is defined as the start of a period lasting at least 0.5 s during which the EMG does not exceed 2 μV above resting EMG. Bilateral leg movements separated by less than 5 s between movement onsets are counted as a single leg movement. Leg movements occurring during a period from 0.5 s preceding a respiratory event to 0.5 s following are not scored according to AASM criteria. Periodic limb movements (PLM) are defined as repetitive leg movements lasting from 0.5 to 10 s, separated by an inter-movement interval ranging from 5 to 90 s, organized in series of at least 4 leg movements. According to the American Academy of Sleep Medicine (AASM), a PLMS index (PLMI) of ≥ 15 was considered clinically significant^36^.

### Statistics

Statistical analyses were performed with SPSS 26.0 for mac. The summary of descriptive statistics was presented as mean (with SD) for continuous variables and as frequencies (with percentages) for categorical variables. ANOVA was used for comparison of quantitative data. Univariate and multivariate logistic regression analyses were conducted to identify associated factors of PLMS among OSAS patients. Statistically significant difference was considered if *P* value < 0 0.05^37^.

The study was reviewed and approved by the Ethics Committee of Sichuan Academy of Medical Sciences & Sichuan Provincial People's Hospital, and conducted in accordance with the Declaration of Helsinki. The approval protocol number of the research ethics committee was 2016LY12.

### Ethics approval and consent to participate

The research was approved by the Ethics Committee of Sichuan Academy of Medical Sciences & Sichuan Provincial People's Hospital. Written informed consent was obtained from all the participants.

### Consent to publish

Written informed consent was obtained from all participants for the publication of any potentially identifiable images or data included in this article.

## Data Availability

The data used and analyzed during the current study are available from the corresponding author on reasonable request.

## References

[1] Medicine., A. A. o. S (2014). American Academy of Sleep Medicine.

[2] Montplaisir J, Michaud M, Denesle R, Gosselin A (2000). Periodic leg movements are not more prevalent in insomnia or hypersomnia but are specifically associated with sleep disorders involving a dopaminergic impairment. Sleep Med..

[3] Mahowald MW (2001). Assessment of periodic leg movements is not an essential component of overnight sleep study. Am. J. Respir. Crit. Care Med..

[4] Karadeniz D, Ondze B, Besset A, Billiard M (2000). Are periodic leg movements during sleep (PLMS) responsible for sleep disruption in insomnia patients?. Eur. J. Neurol..

[5] Jambhekar SK (2011). Periodic limb movements during sleep in children with narcolepsy. J. Clin. Sleep Med..

[6] Fantini ML, Michaud M, Gosselin N, Lavigne G, Montplaisir J (2002). Periodic leg movements in REM sleep behavior disorder and related autonomic and EEG activation. Neurology.

[7] Olson EJ, Boeve BF, Silber MH (2000). Rapid eye movement sleep behaviour disorder: Demographic, clinical and laboratory findings in 93 cases. Brain.

[8] Manconi M (2018). Periodic limb movements during sleep in stroke/TIA: Prevalence, course, and cardiovascular burden. Neurology.

[9] Hermann W (2020). Asymmetry of periodic leg movements in sleep (PLMS) in Parkinson's disease. J. Parkinsons Dis..

[10] Wetter TC, Collado-Seidel V, Pollmacher T, Yassouridis A, Trenkwalder C (2000). Sleep and periodic leg movement patterns in drug-free patients with Parkinson's disease and multiple system atrophy. Sleep.

[11] Sforza E, Juony C, Ibanez V (2002). Time-dependent variation in cerebral and autonomic activity during periodic leg movements in sleep: Implications for arousal mechanisms. Clin. Neurophysiol..

[12] Ferri R (2008). Age-related changes in periodic leg movements during sleep in patients with restless legs syndrome. Sleep Med..

[13] Schipper MH (2020). Sleep-related leg movements in obstructive sleep apnea: Definitions, determinants, and clinical consequences. Sleep Med..

[14] Pennestri MH (2013). Blood pressure changes associated with periodic leg movements during sleep in healthy subjects. Sleep Med..

[15] Pepin JL (2014). Hypertension and sleep: Overview of a tight relationship. Sleep Med. Rev..

[16] Cassel W, Kesper K, Bauer A, Grieger F, Schollmayer E, Joeres L, Trenkwalder C (2016). Significant association between systolic and diastolic blood pressure elevations and periodic limb movements in patients with idiopathic restless legs syndrome. Sleep Med..

[17] Alessandria M, Provini F (2013). Periodic limb movements during sleep: A new sleep-related cardiovascular risk factor?. Front. Neurol..

[18] Bara-Jimenez W, Aksu M, Graham B, Sato S, Hallett M (2000). Periodic limb movements in sleep: State-dependent excitability of the spinal flexor reflex. Neurology.

[19] Giannini G, Zanigni S, Melotti R, Gögele M, Provini F, Facheris MF, Cortelli P, Pramstaller PP (2014). Association between restless legs syndrome and hypertension: A preliminary population-based study in South Tyrol, Italy. Eur. J. Neurol..

[20] Zanigni S, Calandra-Buonaura G, Giannini G, Tonon C, Cortelli P, Provini F (2015). The association between restless legs syndrome, cardiovascular and metabolic diseases: Hypotheses and evidence from the literature. Arch. Ital. Biol..

[21] Association., A. S. D (1997). International Classification of Sleep Disorders, Revised: Diagnostic and Coding Manual.

[22] Haba-Rubioa J, Staner L, Kriegerb J, Machera JP (2005). Periodic limb movements and sleepiness in obstructive sleep apnea patients. Sleep Med..

[23] Cluydts R, De Valck E, Verstraeten E, Theys P (2002). Daytime sleepiness and its evaluation. Sleep Med. Rev..

[24] Ohayon MM, Roth T (2002). Prevalence of restless legs syndrome and periodic limb movement disorder in the general population. J. Psychosom. Res..

[25] Roizenblatt S, Roizenblatt M, Filho FP, Matsuda SS, Mecabo G, Arruda MMAS, Tufik S, Figueiredo MS (2011). Gender difference in periodic limb movements in adults with sickle cell disease. Blood.

[26] Scofield H, Roth T, Drake C (2008). Periodic limb movements during sleep: Population prevalence, clinical correlates, and racial differences. Sleep.

[27] Morrish E, King M, Pilsworth SN, Shneerson JM, Smith IE (2002). Periodic limb movement in a community population detected by a new actigraphy technique. Sleep Med..

[28] Johns MW (1991). A new method for measuring daytime sleepiness: The Epworth sleepiness scale. Sleep.

[29] Zhang Z (2020). Gender differences in clinical manifestations and polysomnographic findings in Chinese patients with obstructive sleep apnea. Sleep Breath.

[30] Shao C (2019). Clinical features and contributing factors of excessive daytime sleepiness in chinese obstructive sleep apnea patients: The role of comorbid symptoms and polysomnographic variables. Can. Respir. J..

[31] Chen NH (2002). Validation of a Chinese version of the Epworth sleepiness scale. Qual. Life Res..

[32] Liu Y (2011). NREM-AHI greater than REM-AHI versus REM-AHI greater than NREM-AHI in patients with obstructive sleep apnea: Clinical and polysomnographic features. Sleep Breath.

[33] Xu M, Yang Y, Zhang J (2013). Levels of neuroglobin in serum and neurocognitive impairments in Chinese patients with obstructive sleep apnea. Sleep Breath.

[34] Kushida CA (2005). Practice parameters for the indications for polysomnography and related procedures: An update for 2005. Sleep.

[35] Berry RB, Brooks R, Gamaldo CE, Harding SM, Marcus CL, Vaughn BV, The American Academy of Sleep Medicine (2012). The AASM Manual for the Scoring of Sleep and Associated Events: Rules, Terminology and Technical Specifications, Version 2.

[36] Medicine., A. A. o. S (2005). International Classification of Sleep Disorders-Diagnostic and Coding Manual.

[37] Zhou X (2020). Risk factors associated with the severity of obstructive sleep apnea syndrome among adults. Sci. Rep..

